# Sensorimotor distance: A grounded measure of semantic similarity for 800 million concept pairs

**DOI:** 10.3758/s13428-022-01965-7

**Published:** 2022-09-21

**Authors:** Cai Wingfield, Louise Connell

**Affiliations:** 1 Department of Psychology, Fylde College, Lancaster University, Lancaster, LA1 4YF UK; 2 Department of Psychology, Maynooth University, Maynooth, Co. Kildare Ireland

**Keywords:** Sensorimotor distance, Semantic similarity, Semantic distance, Grounded cognition

## Abstract

Experimental design and computational modelling across the cognitive sciences often rely on measures of semantic similarity between concepts. Traditional measures of semantic similarity are typically derived from distance in taxonomic databases (e.g. WordNet), databases of participant-produced semantic features, or corpus-derived linguistic distributional similarity (e.g. CBOW), all of which are theoretically problematic in their lack of grounding in sensorimotor experience. We present a new measure of *sensorimotor distance* between concepts, based on multidimensional comparisons of their experiential strength across 11 perceptual and action-effector dimensions in the Lancaster Sensorimotor Norms. We demonstrate that, in modelling human similarity judgements, sensorimotor distance has comparable explanatory power to other measures of semantic similarity, explains variance in human judgements which is missed by other measures, and does so with the advantages of remaining both grounded and computationally efficient. Moreover, sensorimotor distance is equally effective for both concrete and abstract concepts. We further introduce a web-based tool (https://lancaster.ac.uk/psychology/smdistance) for easily calculating and visualising sensorimotor distance between words, featuring coverage of nearly 800 million word pairs. Supplementary materials are available at https://osf.io/d42q6/.

## Introduction

Semantic similarity is at the heart of many fundamental processes in human cognition (see Goldstone & Son, 2012; Hahn, 2014), such as categorisation (e.g. Hampton, 1998; Nosofsky, 1986), memory recall and recognition (e.g. Baddeley, 1966; Montefinese et al., 2015), and language processing (e.g. Hutchison et al., 2008; Raveh, 2002). As semantic similarity between concepts is known to have such wide-ranging effects, accurate and interpretable measures of similarity are crucial tools for predicting behaviour and mapping how people conceptualise and process their world. Thus, such measures are of the utmost utility in research in the cognitive sciences, from designing and analysing experiments to building computational models.

The study of semantic similarity is inseparable from theories of conceptual processing and representation in general. For theories that assume the conceptual system is organised in a taxonomic hierarchy (e.g. Collins & Quillian, 1969; Jolicoeur et al., 1984), natural candidates for measures of semantic similarity may be derived from measures of distance in hierarchical databases (e.g. WordNet, Princeton University, 2010). On the other hand, for family-resemblance accounts in which conceptual relationships are founded on shared features (Rosch & Mervis, 1975; Wittgenstein, 1953; see also Cree & McRae, 2003), measures of semantic similarity can be produced by comparing lists of conceptual features produced in norming studies (e.g. Buchanan et al., 2019; McRae et al., 2005). Under the distributional hypothesis—that similarity of word meaning is given by similarity of word usage (Firth, 1957; Harris, 1954)—linguistic distributional measures of semantic similarity may be derived by extracting relevant statistics from large corpora of natural language (e.g. latent semantic analysis, LSA: Landauer & Dumais, 1997; continuous bag of words, CBOW: Mikolov et al., 2013). Finally, within a grounded cognition framework, where concepts’ representations involve partial replay of perception and action experience (e.g. Barsalou, 1999; Connell & Lynott, 2014), one might surmise that similarity between concepts equates to similarity of their sensorimotor experience; however, no measure of semantic similarity based on sensorimotor experience has been made available to date.

Our goal in the present paper is to address that gap by providing a database of semantic similarity measures based on the sensorimotor experience underlying each concept, which we term *sensorimotor distance* (available online at https://lancaster.ac.uk/psychology/smdistance).

### Measures of semantic similarity

While different measures of semantic similarity have tended to emerge from different (and often conflicting) theoretical traditions, it does not mean they are mutually exclusive. Similarity is a multifaceted and complex construct. For instance, if two things are similar because they share properties in common, then similarity itself is meaningless because all objects share an infinite number of properties in common (e.g. a *plum* and a *lawnmower* both share the properties of weighing less than 100 kg, and less than 101 kg, and less than 102 kg, etc.: Goodman, 1972). Similarity is thus only meaningful when it is constrained to mean two things are similar *in a certain respect*, and it is possible that multiple measures of similarity, each applying different constraints, are required to fully capture the similarity between two given concepts.

Similarity measures based on hierarchical structure can be taken from large machine-searchable encyclopaedic databases (e.g. Strube & Ponzetto, 2006), or purpose-built semantic databases such as WordNet (Miller, 1995, 1998; Princeton University, 2010). WordNet is a large online lexical database of English, with words organised into a hierarchy of hypernymic (i.e. “is a type of”) relations. Under this framework, concepts are more similar when there is a short path between their nodes in the hierarchical structure (Jiang & Conrath, 1997; Resnik, 1995). For example, similarity measures based on WordNet distance are likely to score *alligator* and *crocodile* as highly similar because the path between them is very short (e.g. *alligator → crocodilian reptile → crocodile*), but will score *alligator* and *monster* as quite dissimilar because the path between them requires going via the root node of *entity* and is thus very long indeed^1^. Coverage of similarity comparisons using WordNet distance is very high in principle (i.e. over 117,000 synset classes potentially enables billions of pairwise comparisons), although it is limited to separate consideration of nouns and verbs because other parts of speech are not structured in hypernymic hierarchies; “off-the-shelf” coverage is far smaller in reality, such as Maki et al.’s (2004) compilation of WordNet distances for nearly 50,000 concept pairs. However, the nature of hierarchical distance as a similarity measure means that while it excels at constraining similarity by hypernymic/categorical relations, the role of sensorimotor grounding is largely non-existent. While concepts very close together in hierarchical structure may share some sensorimotor experience (e.g. many types of *foodstuff* may be grounded in taste and smell), other forms of semantic similarity that are grounded in perceptual or action resemblances (e.g. *alligator* and *monster*; *princess* and *bride*; *toddler* and *detonation*) are not generally captured.

Feature-based similarity measures, on the other hand, are typically computed from lists of features produced by participants per concept in norming studies. Under this framework, similarity between a pair of concepts is given by the degree of overlap of their respective lists of features. Feature lists are necessarily highly sparse (i.e. most concepts do not possess most features); overlap can therefore be determined by simple counting of common features (McRae et al., 2005), or incorporating feature-production frequencies (e.g. by using the cosine of the angle between feature-frequency vectors: Devereux et al., 2014; Buchanan et al., 2019). For example, the concepts *mountain* and *hill* would be scored as similar because they have many shared features such as *high*, *landscape, climb*, and *steep*, whereas *mountain* and *pyramid* would be far less similar because they share far fewer features (e.g. *tall*). By encompassing a wide range of features including taxonomic (e.g. *landscape*), encyclopaedic (e.g. *found in ranges*), and grounded (e.g. *cold*), feature-based measures can theoretically constrain similarity on a number of different dimensions. However, grounded features are not consistently present across concepts (e.g. *toy* has no perceptual or action features in McRae et al.’s norms; *music* has no action features in Buchanan et al.’s norms), and so a measure of semantic similarity based on concept-feature norms is, at best, inconsistently and partially grounded. In addition, the laborious nature of collecting and standardising feature lists produced by participants has meant that feature-based similarity measures are quite restricted in their coverage. One of the largest concept-feature norming studies is that of Buchanan et al. (2019), who compiled a database of features for almost 4500 concepts that expanded on several previous databases (including Devereux et al., 2014; McRae et al., 2005; Vinson & Vigliocco, 2008), and made available feature-based similarity measures for over 200,000 concept pairs. While useful, feature-based measures nonetheless cover only a small fraction of the approximately 40,000 concepts thought to make up the typical conceptual system of adult English speakers (Lynott et al., 2020; see also Brysbaert et al., 2014) and a smaller fraction of the hundreds of millions of comparable concept pairs. Moreover, since many concept-feature norming studies focused exclusively on concrete noun concepts, particularly objects (e.g. Devereux et al., 2014; McRae et al., 2005), and later studies expanded those item sets (Buchanan et al., 2019), abstract concepts and other parts of speech remain underrepresented in feature-based similarity measures.

Finally, linguistic distributional measures of semantic similarity are based on the statistical relationships between words and their usage contexts in natural language. Under this framework, similarity of concepts is determined by contextual similarity of their word labels, following the distributional hypothesis that words with similar meanings tend to occur in similar contexts (Harris, 1954). Linguistic distributional measures of semantic similarity were recently typified by Mikolov et al.’s (2013) continuous bag of words (CBOW), which represents words as vectors derived from a neural network model trained on word co-occurrences in a corpus of text; similarity between two concepts is then compared as the cosine similarity between these vectors. For example, CBOW scores *helicopter* and *airplane* as highly similar because they appear in similar contexts (e.g. concerning *pilot, flying, sky*), but scores *helicopter* and *bee* as dissimilar because they tend to occur in quite different contexts. Other examples of linguistic distributional measures include latent semantic analysis (LSA: Landauer & Dumais, 1997, which continues to be used extensively in the cognitive sciences as a measure of semantic similarity), GloVE (Global Vectors for Word Representation, Pennington et al., 2014), and skip-gram (Mikolov et al., 2013: CBOW’s sister model in the word2vec package). Linguistic distributional measures of semantic similarity have excellent coverage, with tens or hundreds of thousands of individual words available for comparison (depending on the corpus) that span all parts of speech. They also appear to constrain similarity on a number of different dimensions, such as synonymity, shared categories, taxonomic classes, and thematic connections (see Wingfield & Connell, 2022, for review). However, linguistic distributional measures can approximate sensorimotor grounding only insofar as this information is reflected in statistical patterns of word usage, which is limited. For example, Louwerse and Connell (2011) showed that language-use statistics were able to distinguish visuohaptic words from auditory words, but not visual words from haptic (see also Louwerse & Jeuniaux, 2008; Riordan & Jones, 2011). In general, linguistic distributional measures do not capture many forms of semantic similarity that are grounded in perceptual or action resemblances^2^ (e.g. *helicopter* and *bee*, *toddler* and *detonation*).

### The current norms: Sensorimotor distance

We present here a novel, grounded measure of semantic similarity: *sensorimotor distance*. It is based on the Lancaster Sensorimotor Norms (Lynott et al., 2020), which contain sensorimotor strength ratings that reflect the extent to which a given referent concept can be perceived through auditory, gustatory, haptic, interoceptive, olfactory, and visual modalities; or can be experienced by performing an action with the hand/arm, head, foot/leg, mouth, or torso effectors. Each of these dimensions was carefully chosen to map to a specific, separable region of the cortex, meaning that a multidimensional profile of sensorimotor strength approximates the distributed neural representation of a concept across the sensory, insular, and motor cortices, and hence operationalises how the perception and action systems provide distributed grounding for words. Each concept is represented as a point (or vector) in an 11-dimensional space of distributed sensorimotor experience, and distance between concepts can therefore be calculated as the distance between the vectors. For example, *alligator* and *monster* are relatively close in sensorimotor terms (i.e. both are experienced primarily by sight, moderately by hearing and head action, weakly by touch and hand action; but are not generally smelled or involve action with the mouth, foot, or torso), whereas *alligator* and *daydream* are quite distant because they share little sensorimotor experience.

Sensorimotor distance is therefore a grounded measure of semantic similarity that operationalises how the distributed neural representations of two concepts across perception and action systems differ from one another^3^. Its coverage is excellent, as the set of nearly 40,000 concepts in the Lancaster Sensorimotor Norms is large enough to approximate a full adult conceptual system, covering both abstract and concrete concepts and all parts of speech, yielding nearly 800 million comparable concept pairs.

Sensorimotor distance constrains similarity by perception and action experience, and by its nature would also constrain by synonymity (i.e. synonyms like *sofa* and *couch*, or *large* and *big*, would be expected to have extremely similar profiles of sensorimotor experience). Recent work in our lab also suggests that sensorimotor distance appears to capture taxonomic/categorical constraints. For instance, sensorimotor distance between category name and member concept has been successfully used to predict responses in category production (e.g. list as many types of *animal* as you can: Banks et al., 2021) and category verification tasks (e.g. is the pictured *dog* a member of the category *animal*?: van Hoef et al., 2019). Participants were more likely to list a member concept as belonging to a category, and to verify its membership quickly and accurately, when it had short sensorimotor distance from the category concept (e.g. *animal* and *dog*) compared to longer sensorimotor distance (e.g. *animal* and *snake)*. Nonetheless, sensorimotor distance would not generally capture all forms of semantic similarity, such as those based on thematic relationships between concepts (e.g. *bee* and *honey*; *grape* and *vineyard*).

In the current paper, we present the details of the sensorimotor distance measure, and demonstrate that sensorimotor distance has comparable explanatory power to WordNet distance, feature overlap, and CBOW in modelling human similarity judgements while explaining variance in human judgements that is missed by other measures. Furthermore, it does so with the advantages of remaining both grounded and computationally efficient (i.e. easy to calculate via economical representations, once the relevant sensorimotor ratings have already been collected), and applies to both abstract and concrete concepts. All data, analysis code, and full results are available in supplemental materials at https://osf.io/d42q6/. We further introduce a web-based tool (available at https://lancaster.ac.uk/psychology/smdistance) for easily calculating and visualising sensorimotor distance between lists of concepts, featuring coverage of nearly 800 million concept pairs.

## Calculating sensorimotor distance

### Materials

We took all 39,707 concepts from Lynott et al.’s (2020) Lancaster Sensorimotor Norms, which provide ratings along 11 dimensions of sensorimotor experience as well as a number of other related variables. Lynott et al. normed perceptual and action dimensions separately on a total of 3500 native speakers of English. For the perceptual norming (*N* = 2635), participants were asked to rate on a scale from 0 (not at all) to 5 (greatly) to what extent they experienced a concept by seeing, hearing, feeling through touch, sensations inside the body, smelling, and tasting (six perceptual modalities, randomly ordered). For the action norming (*N* = 1933), participants were asked to rate on the same scale to what extent they experienced a concept by performing an action with the hand/arm, foot/leg, head excluding mouth, mouth/throat, and torso (5 action effectors, each accompanied by a body avatar image for clarity, randomly ordered). Participants could select a “don’t know” button instead of providing ratings when they were not familiar with the named concept. The final dataset comprised 12.3 million individual ratings and showed excellent inter-rater reliability for all dimensions (Cronbach’s alpha = .85–.96). We use here the main form of the norms at the item level, which comprise mean ratings per dimension for 39,707 concepts.

### Measures of sensorimotor distance

To compute sensorimotor distance between a pair of concepts, we use the vectors of ratings in each of the 11 dimensions of sensorimotor experience. Many possible measures exist for calculating the distance between vectors; here we present *cosine distance* (i.e. 1 minus the cosine of the angle between the vectors^4^), which we found to be the best for modelling human similarity judgements. We also tested four other examples: correlation, Euclidean, Minkowski-3, and Mahalonobis distances^5^, with details included in the supplementary materials. Any pair of concepts in the Lancaster Sensorimotor Norms can be compared using cosine distance, yielding sensorimotor distance scores for over 788 million unique concept pairs.

### Sensorimotor distance characteristics

Sensorimotor distance computations between concept pairs, and other associated functions such as finding nearest neighbours and plotting two-dimensional visualisations, can be performed using an online tool at https://lancaster.ac.uk/psychology/smdistance, detailed in Appendix 1.

#### Distance distributions

**Fig. 1:**
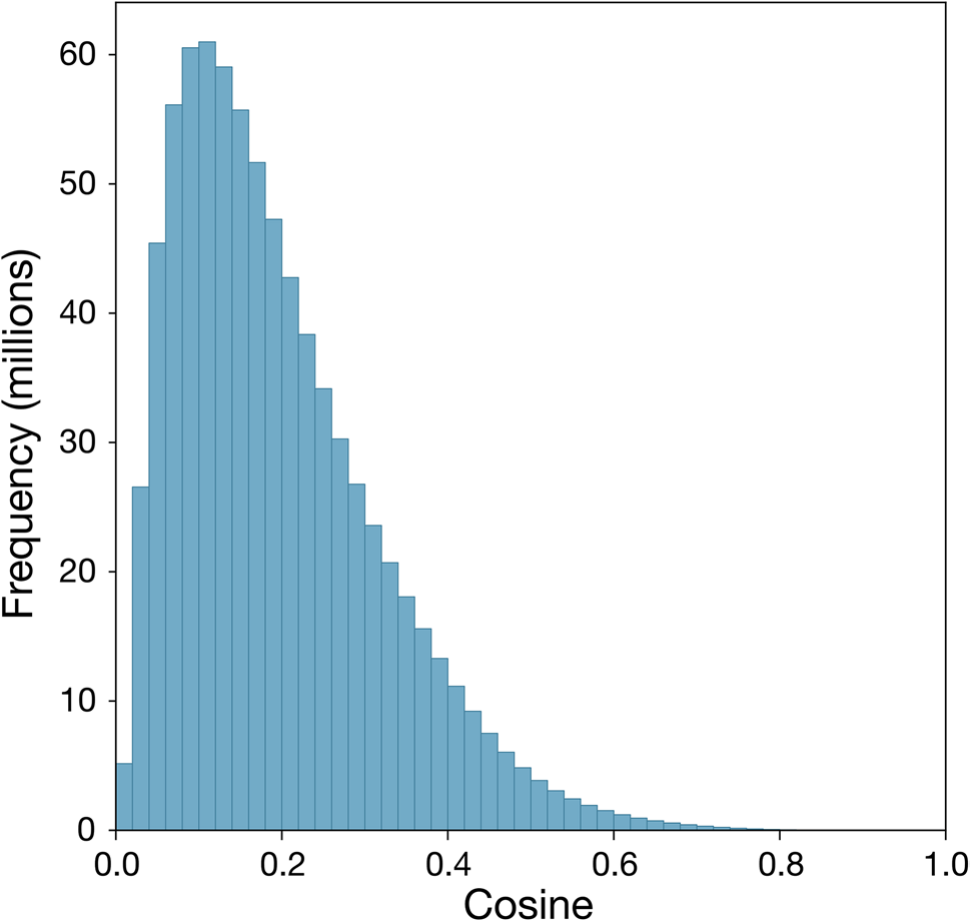
Distributions of cosine distances between all (approximately 800 million) pairs of concepts in the Lancaster Sensorimotor Norms

Cosine distances between non-negative vectors range in theory from 0 to 1, and sensorimotor distance measures span almost the entire range of possible values: the minimum attained distance is .0002 (the closest pair is *cyan–pixilation*, with other very close pairs including *hyphen–colorfast*, distance 0.0020, and *everything–multisensory*, distance 0.0038; excluding the distances of zero between each concept and itself) and the maximum is .950 (the furthest pair is *shinbone–smelled*, with other very distant pairs including *flavorless–handgrip*, distance 0.942, and *adobe–digestion*, distance 0.921). The full distribution of distances is shown in Fig. 1. Mean sensorimotor distance was .195 (*SD* = .123).

#### Visualizing distance between concepts

**Fig. 2:**
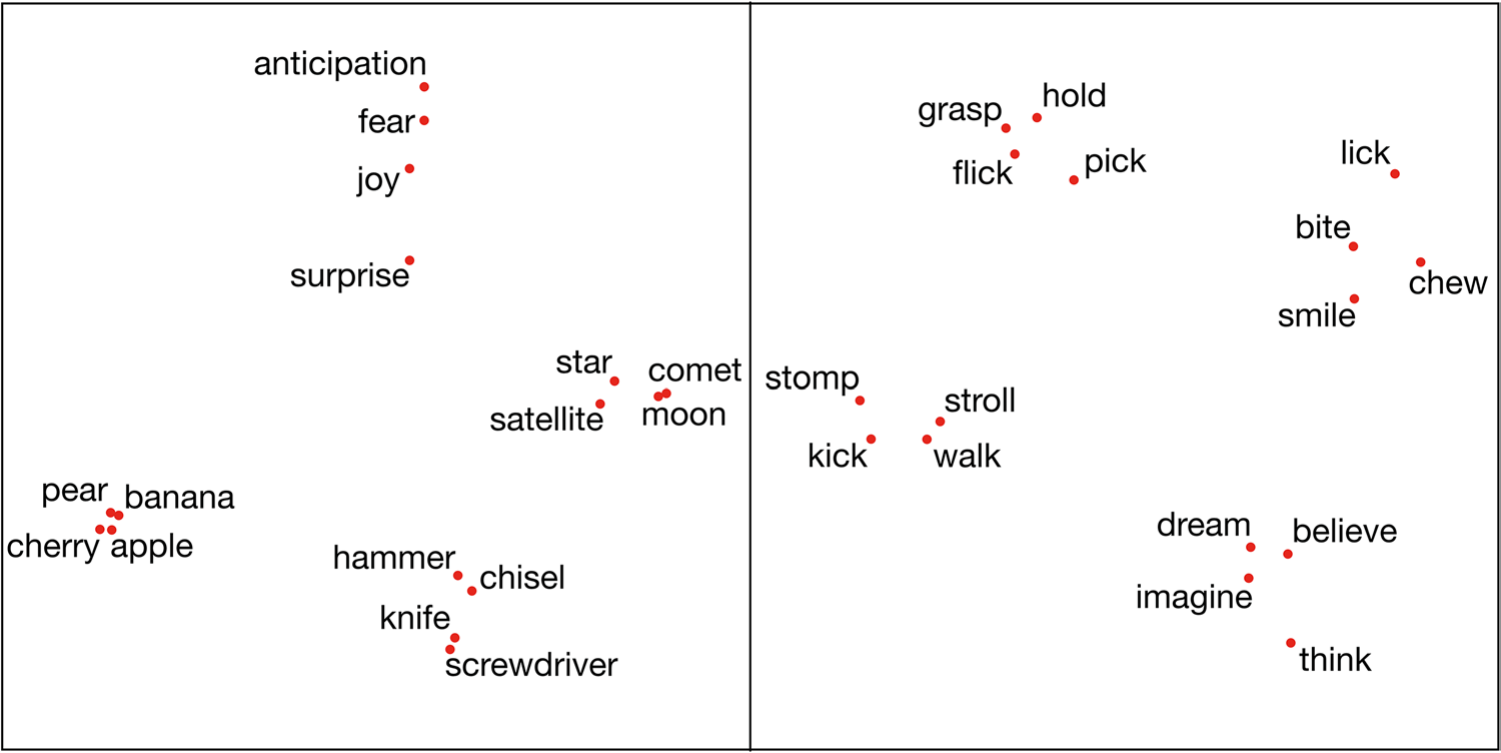
Visualizing sensorimotor distance between sample concepts. Cosine distances between each pair of concepts were transformed using nonmetric multidimensional scaling (Sammon, 1969). Left panel: select nouns for tools, emotions, fruit, and celestial objects. Right panel: select verbs for leg, hand, mouth, and cognitive actions

The relative distances between select concepts can be visualised using multidimensional scaling (MDS) techniques, which arrange points in two-dimensional space while minimizing the distortion of the pairwise distances. Figure 2 shows two examples of such MDS plots for a selection of category exemplars taken from the norms, demonstrating clustering between semantic categories of nouns and action categories of verbs (see also Connell et al., 2019).

#### Nearest neighbours

**Fig. 3:**
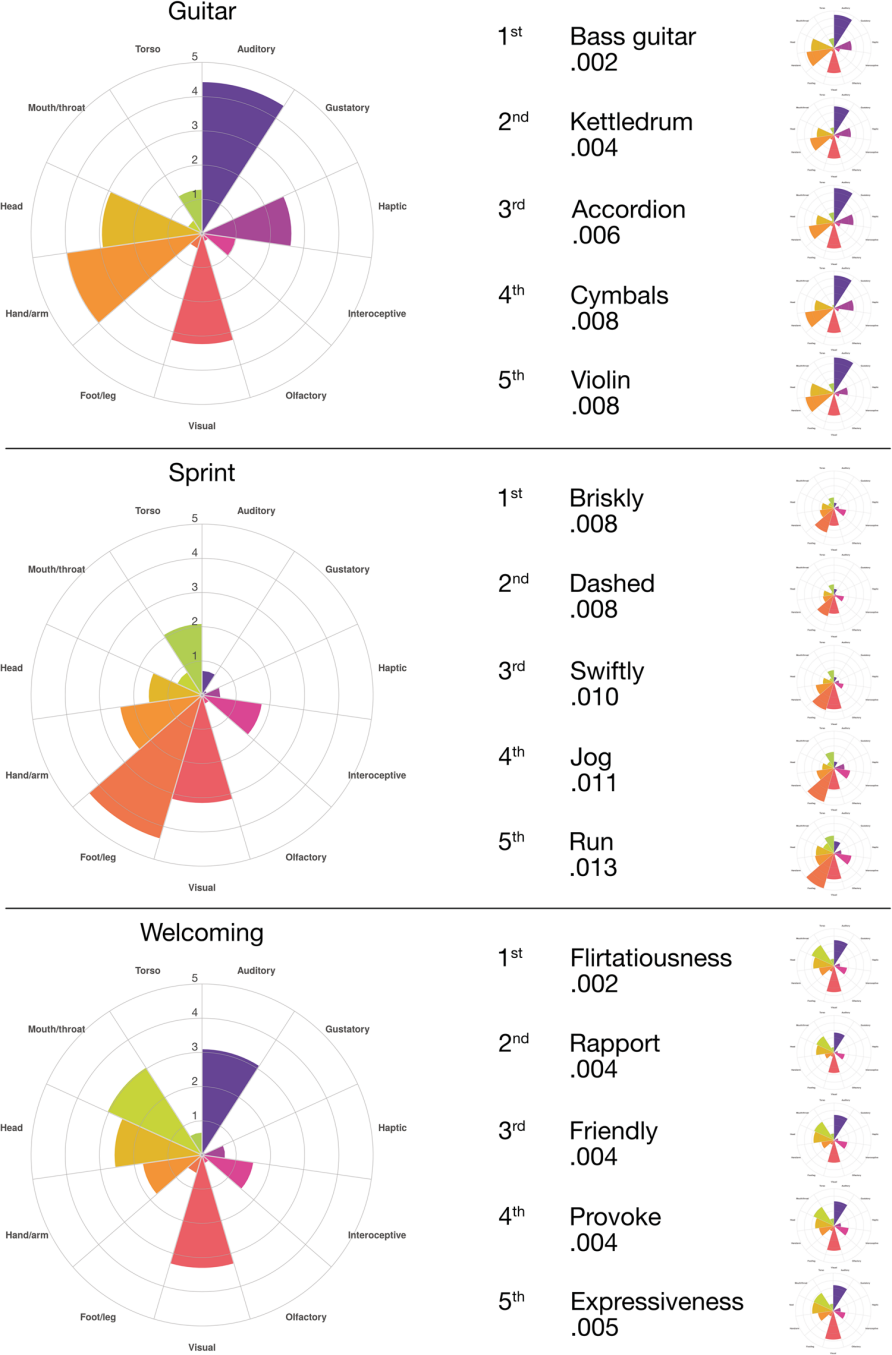
*Examples of top*-*five nearest neighbo*urs in sensorimotor distance. Each concept is accompanied by a polar plot, which shows the strength of rating in each dimension: (clockwise from the top) auditory, gustatory, haptic, interoceptive, olfactory, visual, foot/leg, hand/arm, head, mouth/throat, torso

From a reference word, lists of nearest sensorimotor neighbours (i.e. the other concepts which have the smallest distance to the reference word) can be generated. Some examples of nearest neighbours are shown in Fig. 3, suggesting that sensorimotor distance can encode detailed information about concepts (e.g. speed of movement).

## Validating sensorimotor distance

For sensorimotor distance to be a useful research tool, it is important to show both how it compares to other measures of semantic similarity, and that it is a good predictor of human judgements of similarity which are missed by other measures. All materials, data, and associated statistics are available in the supplementary materials at https://osf.io/d42q6/.

### Analysis 1: Comparison to other measures of semantic similarity

In this first analysis of convergent validity, we compare sensorimotor distance as a grounded measure of semantic similarity with alternative similarity measures that originate in different theoretical perspectives on the conceptual system: hierarchical structure (i.e. WordNet distance), feature-based representations (i.e. feature overlap), and linguistic distributional information (i.e. CBOW). Overall, sensorimotor distance correlates as well with alternative measures of semantic similarity as such measures do with each other.

#### Method and materials

 We compiled 4325 word pairs featured in existing datasets of human similarity ratings: WordSim (Finkelstein et al., 2002), Simlex (Hill et al., 2016), and MEN (Bruni et al., 2014). Coverage varied by measure, as outlined below.

In addition to our own sensorimotor distance measure, we selected three popular measures of semantic similarity which have been widely used across the cognitive sciences, each relating to one of the theoretical frameworks discussed earlier:

##### Sensorimotor distance

A total of 3730 word pairs were covered by our database, for which we calculated sensorimotor distance (cosine distance *M* = .126 *SD* = .104).

##### WordNet distance

Maki et al. (2004) compared several related measures based on distance in the WordNet taxonomy, from which the authors determined that Jiang–Conrath distance (Jiang & Conrath, 1997) was the superior choice for modelling semantic similarity. Jiang–Conrath distance is based on the information content of two concepts relative to that of their most specific mutual ancestor in the hierarchy (i.e. the “least common subsumer”). Although Maki et al. make available a database of precomputed distances for around 50,000 word pairs, it covers only approximately 10–15% of most of the similarity datasets we set out to model here. We therefore opted to recompute Jiang–Conrath distances on WordNet using the implementation in NLTK (Natural Language Toolkit) version 3.2 (Bird et al., 2009), which covered 3776 word pairs (WordNet distance *M* = 11.39, *SD* = 6.06).

##### Feature overlap

Buchanan et al. (2019) collected feature-production norms for a list of 4436 concepts. Pairs of concepts can be compared via their respective lists of norms^6^. Instead of counting the number of norms in common between a concept pair, Buchanan et al. recommend computing the cosine of the angle between the sparse property-frequency vectors (yielding approximately 10 million comparable pairs). Buchanan et al. provide a database of precomputed cosine-overlap values for just over 208,000 pairs, which covered 2414 pairs from our item set (feature overlap *M* = .095, SD = .189).

##### CBOW

The computation of CBOW scores involves training a neural network model on a huge corpus of text to predict a target word from its linguistic contexts (Mikolov et al., 2013). We used the CBOW vectors from Mandera et al. (2017, provided by Mandera, 2016) to calculate cosine distances for our materials: 4325 word pairs were covered (CBOW cosine distance *M* = .693, *SD* = .162).

#### Analysis

 We computed Bayesian correlations between all four semantic similarity measures using JASP (JASP Team, 2020) with a stretched prior beta width = 1 (i.e. uniform prior where all correlations values are equally likely). Because some similarity measures were distances (i.e. more similar = lower score) while others were similarity/overlap scores (i.e. more similar = higher score), the direction of the alternative hypothesis varied. Matching constructs were expected to be positively correlated (i.e. between sensorimotor distance, WordNet, and CBOW), whereas mismatching constructs (i.e. all other comparisons) were expected to be negatively correlated. Bayes factors (BF) are reported as natural logarithms due to their magnitude.

#### Results

**Fig. 4:**
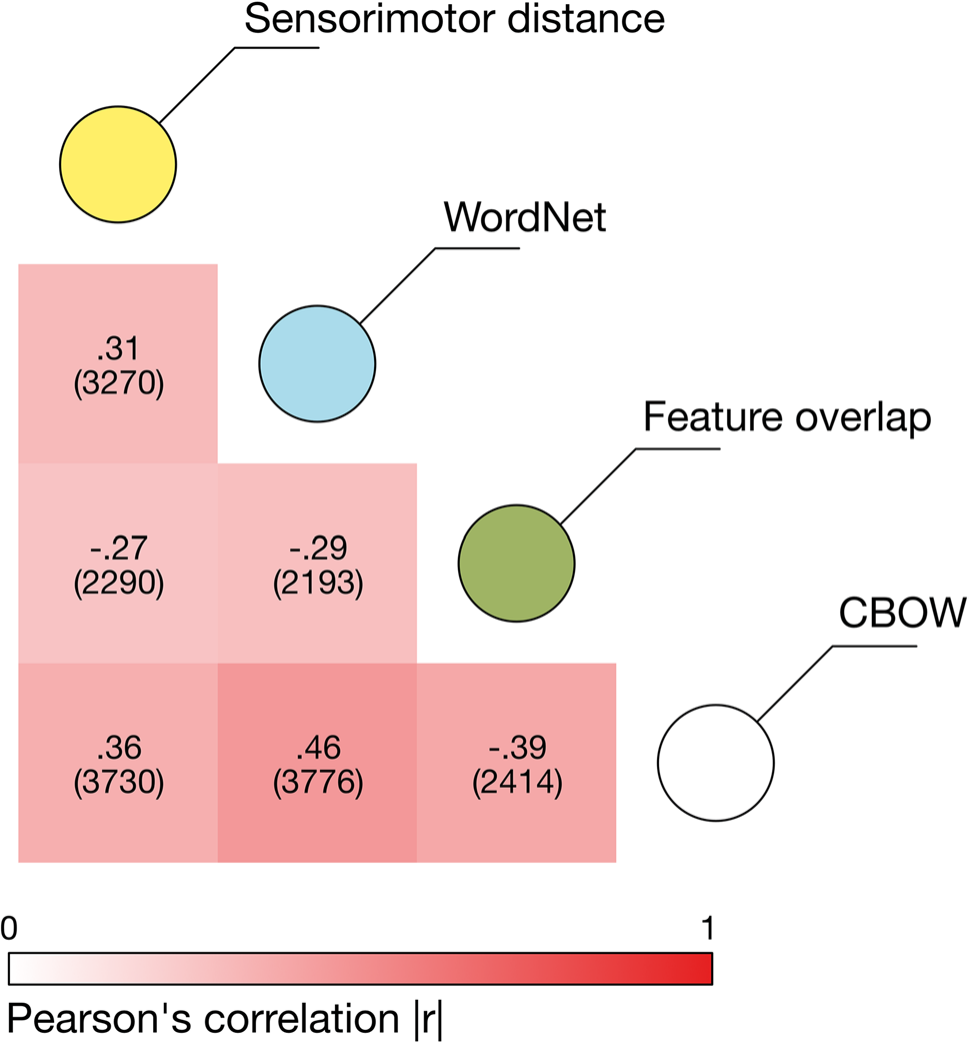
Correlations between sensorimotor distance and three other measures of semantic similarity. The colour scale indicates the absolute value of the correlation (i.e. stronger colour = stronger relationship) while the correlation sign varies according to whether the variable is a measure of distance or similarity/overlap. The number of pairs per comparison is in parentheses (2081 pairs were common to all measures)

 Sensorimotor distance correlated at best moderately with other measures of semantic similarity (see Fig. 4), with very strong evidence that the correlations ran in the expected direction: all log BFs > 80. Intercorrelations between WordNet distance, feature overlap, and CBOW scores were of similar magnitude, indicating that sensorimotor distance correlated with other measures of semantic similarity about as well as they correlate with each other. Full statistics for all comparisons can be found in the supplementary materials.

Sensorimotor distance therefore incorporates unique information that is not captured by other measures of semantic similarity, although it is not yet clear whether this unique information reflects semantic similarity itself as opposed to mere noise. We address this question in the following section by examining its external validity in predicting human similarity judgements.

### Analysis 2: Predicting human similarity judgements

In this section, we demonstrate external validity by examining how effectively sensorimotor distance can predict human judgements of semantic similarity and compare its performance to other measures. Using three different datasets of human similarity judgements, we demonstrate that sensorimotor distance can explain unique variance above and beyond each alternative measure of semantic similarity (i.e. WordNet, feature overlap, CBOW). In addition, given that each measure constrains semantic similarity in a different way that is potentially useful to modelling human data, we examine what combination of semantic similarity measures best explains human similarity judgements. Across the three datasets of human similarity data, we find that sensorimotor distance is consistently included in the best-fitting model and demonstrates the most consistent level of performance.

#### Method and materials

 To compare the relative explanatory power of each model of semantic similarity, we examined participant similarity judgements from three existing datasets: Simlex-999 (Hill, n.d.; Hill et al., 2016: 999 word pairs), WordSim-353 (Gabrilovich, 2002; Finkelstein et al., 2002: 353 word pairs), and MEN (Bruni, 2012; Bruni et al., 2014: 3000 word pairs). In the Simlex and WordSim datasets, participants directly rated the similarity of pairs of words and the dependent variable is the mean similarity rating per word pair. In the MEN dataset, however, participants selected the most closely related out of two possible word pairs in a forced-choice paradigm; these choices were then converted into a single similarity score for each pair. From each dataset, we selected only those items that were covered by all four of the semantic similarity measures, resulting in 669 word pairs from Simlex, 181 from WordSim, and 1251 word pairs from MEN.

#### Analysis

 Each dataset was analysed separately but identically in three stages. We first computed zero-order correlations between the human similarity scores and each of the four semantic similarity measures (i.e. sensorimotor distance, WordNet distance, feature overlap, CBOW); Bayesian correlation was carried out in JASP as described in the previous section.

Next, to examine the independent contribution of sensorimotor distance, we carried out hierarchical Bayesian linear regressions (JASP Team, 2020: using JSZ default priors, r scale = .354, beta binomial distribution a = 1 and b = 1) on human similarity judgements. Step 1 entered one of the other semantic similarity measures (i.e. WordNet distance, feature overlap, or CBOW scores), and Step 2 entered sensorimotor distance. Model comparisons using BF between steps therefore tested whether sensorimotor distance explained unique variance in human similarity judgement above and beyond other similarity measures. In this analysis, log BF for Step 2 over Step 1 is equivalent to the inclusion Bayes factor (BF-inclusion).

Finally, to find the best possible model of human similarity judgements for each of the three datasets, we conducted Bayesian linear regressions (settings as above) by examining all possible combinations of all four semantic similarity measures as predictors, and selecting the model that offered the best fit to that dataset. We also report BF-inclusion for each predictor, which reflects the change from prior to posterior odds for all models including a particular predictor compared to models excluding it (Hinne et al., 2020), and allows us to compare the relative strength of evidence for each similarity measure in predicting each dataset of human similarity judgements.

#### Results

**Fig. 5:**
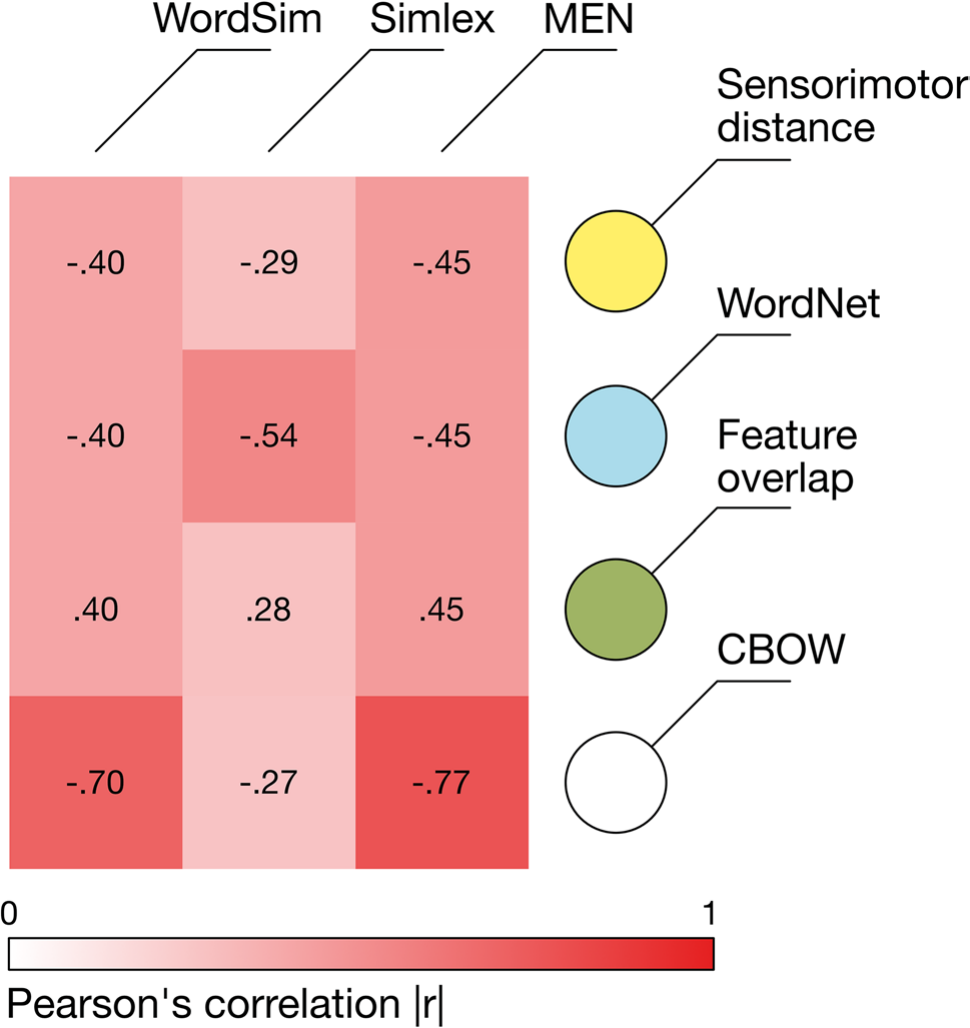
Zero-order correlations between human similarity judgements and each measure of semantic similarity, calculated separately per dataset. The colour scale indicates the absolute value of the correlation (i.e. stronger colour = stronger relationship) while the correlation sign varies according to whether the variable is a measure of distance or similarity/overlap

Figure 5 shows zero-order correlations between each semantic similarity measure and human similarity judgements from each dataset. Sensorimotor distance was moderately correlated with human similarity scores (i.e. shorter distance = more similar), with the magnitude of the correlations within the bounds achieved by alternative similarity measures. All correlations had very strong evidence in the expected direction (log BFs > 13.4).

**Fig. 6:**
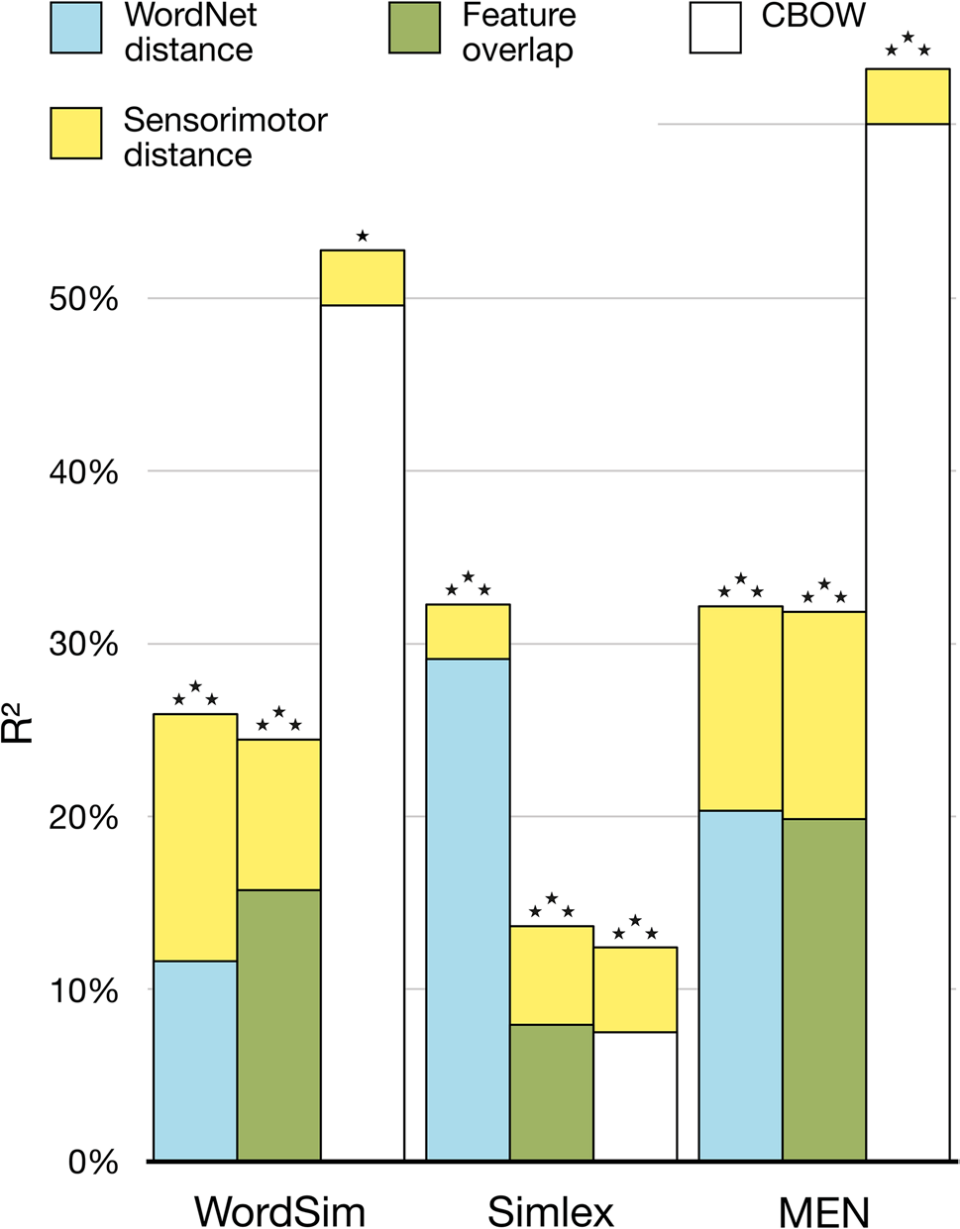
Unique effects of sensorimotor distance (top of stacked bar; yellow) in explaining variance in human similarity judgements when added to a regression model already containing an alternative measure of semantic similarity (bottom of stacked bar; colour varies). Regressions were performed separately for each dataset (WordSim, Simlex, MEN) and alternative measure of semantic similarity (WordNet distance, feature overlap, CBOW). Asterisks indicate evidence for including sensorimotor distance at Step 2 compared to alternative predictor at Step 1 (* log BF_10_> log 10; ** log BF_10_> log 100; *** log BF_10_ > log 1000)

In the hierarchical regression analyses, there was strong evidence for the inclusion of sensorimotor distance at Step 2 in all models: see Fig. 6 for change in *R*^2^ and Table 1 for coefficients. For all three datasets, sensorimotor distance explained variance in human similarity judgements above and beyond that explained by alternative measures of semantic similarity (i.e. WordNet distance, feature overlap, CBOW). In all analyses, variance inflation factors were approximately 1, indicating that multicollinearity was not an issue.

**Table 1:** Regression coefficient statistics for Step-2 models of human similarity judgements across three datasets, showing coefficient estimates and their 95% credible intervals for sensorimotor distance and each alternative semantic similarity measure, as well as natural log of inclusion Bayes factors for the sensorimotor distance predictor

Dataset	Step-1 semantic predictor	Step-2 sensorimotor distance coefficient	95% CI		Log BF-inclusion
			Lower	Upper	
WordSim	WordNet	−7.30	−10.60	−4.69	9.09
	Feature overlap	−6.90	−10.31	−4.21	7.45
	CBOW	−4.35	−7.19	−1.63	3.37
Simlex	WordNet	−5.13	−6.96	−3.35	12.24
	Feature overlap	−6.84	−8.89	−4.84	18.64
	CBOW	−6.50	−8.61	−4.44	15.62
MEN	WordNet	−39.39	−44.65	−34.23	96.72
	Feature overlap	−39.71	−44.97	−34.53	97.99
	CBOW	−21.59	−25.54	−17.59	49.49

Overall, these results indicate that the unique information captured by sensorimotor distance is *not* mere noise. Rather, they suggest that sensorimotor distance constrains similarity in a way that is not captured by other measures of semantic similarity that relate to hierarchical structure (i.e. WordNet distance), feature-based representations (i.e. feature overlap), or linguistic distributional information (i.e. CBOW).

**Fig. 7:**
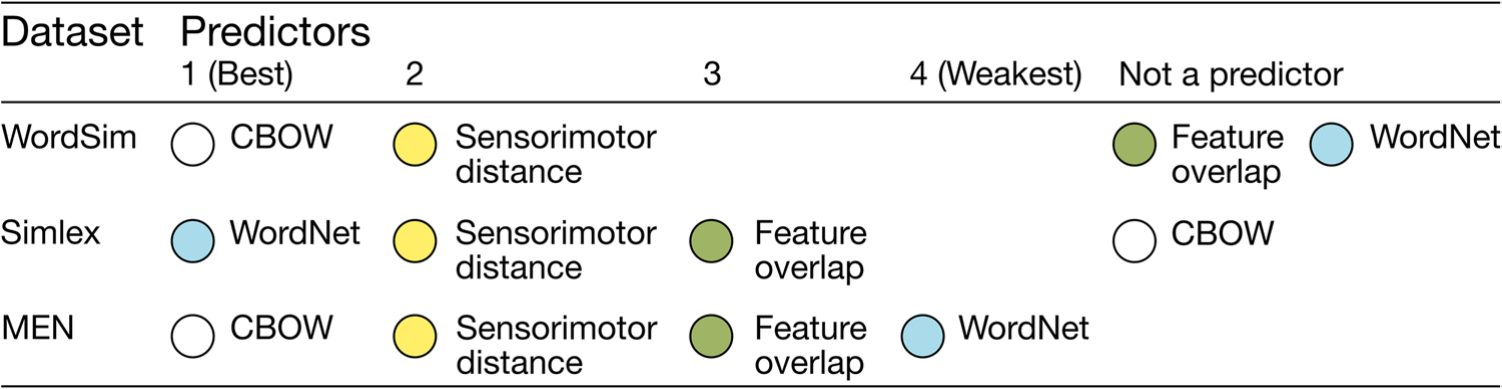
Rank order from best to worst of each semantic similarity measure in predicting human similarity judgements across three datasets, based on inclusion Bayes factors in best-model regressions

Finally, in the best-model regressions, the optimal predictors of human similarity judgement varied by dataset, as did the relative evidence for each predictor (see Fig. 7 for summary and Table 2 for coefficient statistics). For WordSim, only CBOW and sensorimotor distance were included in the best model, which explained over half the variance with a very strong level of evidence (*R*^2^ = .527, log BF_10_ = 60.92; full statistics for all candidate models are available in supplemental materials). Inclusion Bayes factors indicated that CBOW was the best predictor of human similarity judgements in WordSim, followed by sensorimotor distance. Notably, there was evidence *against* including feature overlap as a predictor of human similarity judgements in WordSim (i.e. a model including feature overlap was log BF_10_ = −2.14 times worse than the best model of just CBOW and sensorimotor distance), and no positive evidence for including WordNet distance (i.e. a model including WordNet distance was log BF_10_ = −0.76 times worse than the best model). For Simlex, the best model comprised (in rank order of BF-inclusion) Wordnet distance, sensorimotor distance, and feature overlap, which explained a third of the variance with a very strong level of evidence (*R*^2^ = .338, log BF_10_ = 128.12). In this case, there was evidence against including CBOW (i.e. a model containing all four measures was log BF_10_ = −2.383 times worse than the best model that excluded CBOW), despite it being the best predictor of WordSim similarity. For the MEN dataset, all measures of semantic similarity were included in the best model, which this time explained a very high 65% of variance with a very strong level of evidence (*R*^2^ = .651, log BF_10_ = 642.08). The best predictor by BF-inclusion was CBOW, followed by sensorimotor distance, then feature overlap, and lastly WordNet distance (i.e. the weakest predictor of MEN similarity despite being the best predictor of Simlex similarity).

**Table 2:** Regression coefficient statistics for the most complex model of human similarity judgements across three datasets, showing coefficient estimates and with 95% credible intervals for sensorimotor distance and each alternative semantic similarity measure, as well as natural log of inclusion Bayes factors for each predictor

Dataset	Parameter	Coefficient	95% Credible interval		Log BF-inclusion
			Lower	Upper	
WordSim	Intercept	5.88	5.64	6.07	
	WordNet	−0.04	−0.07	1.49E−4	−0.12 ^a^
	Feature overlap	0.20	−0.43	1.41	−1.17 ^b^
	CBOW	−7.44	−9.35	−6.20	35.62
	Sensorimotor distance	−4.00	−7.19	−1.36	3.24
Simlex	Intercept	4.34	4.18	4.49	
	WordNet	−0.22	−0.25	−0.19	80.19
	Feature overlap	1.47	0.69	2.19	5.54
	CBOW	0.07	−0.57	0.92	−1.00 ^c^
	Sensorimotor distance	−4.54	−6.39	−2.74	9.71
MEN	Intercept	25.45	25.04	25.85	
	WordNet	−0.14	−0.25	−0.05	3.45
	Feature overlap	9.46	6.88	12.00	23.43
	CBOW	−45.93	−49.43	−43.40	341.18
	Sensorimotor distance	−18.87	−22.83	−14.97	40.33

^a^Indicates equivocal evidence *against* inclusion of WordNet scores in model of WordSim dataset

^b^Indicates evidence *against* inclusion of feature overlap scores in model of WordSim dataset

^c^Indicates equivocal evidence *against* inclusion of CBOW scores in model of Simlex dataset

Overall, these best-model regressions showed that no single measure of semantic similarity was consistently preferred as the top predictor of human similarity judgements. Sensorimotor distance was present in every best model, and no other predictor was consistently ranked better across all datasets. On the other hand, sensorimotor distance was never the overall best predictor, and was only consistently preferred to feature overlap over all datasets. We note that the pattern of results changed little when we examined an alternative linguistic distributional model (LSA; see Appendix 2), which suggests that our findings generalise beyond the particular implementation of CBOW (e.g. corpus size can affect performance: see Bullinaria & Levy, 2012; Wingfield & Connell, 2022). This pattern of results is consistent with the idea that different measures of semantic similarity constrain similarity in different ways, all of which are relevant to what humans consider when judging the similarity of concepts.

## Sensorimotor distance for abstract and concrete concepts

As a measure of semantic similarity that is based on perception and action experience, some might wonder whether sensorimotor distance could apply to abstract concepts, which in some accounts are defined by their lack of perceptual information (e.g. Paivio, 1986). Previous research has shown that virtually all concepts, regardless of their concreteness, are experienced to some extent through various sensorimotor dimensions. Connell and Lynott (2012) showed that many abstract concepts tend to be strongly perceptual (i.e. their experience involves perception, particularly vision), Connell et al. (2018) found that interoceptive strength (i.e. sensations inside the body) was *more* important to abstract concepts than to concrete, and Lynott et al.’s (2020) norms demonstrate multidimensional sensorimotor profiles for many abstract concepts such as *justice* and *everything*. In principle, therefore, sensorimotor distance should apply as a measure of semantic similarity between abstract concepts as well as between concrete concepts (see also Fig. 3).

To examine this principle in action, we compared the ability of sensorimotor distance to predict human similarity judgements in three different categories of concept pairs: both concepts abstract (e.g. *inexpensive* and *cheap*), mixed concrete–abstract (e.g. *battle* and *conquest*), and both concepts concrete (e.g. *drizzle* and *rain*).

### Method and materials

Of the three datasets of human similarity judgements examined in validation analysis 2, only one contained sufficient numbers of abstract concepts to enable meaningful comparisons: Simlex-999 (Hill et al., 2016)^7^. Using Brysbaert et al.’s (2014) concreteness ratings, we categorised concepts as abstract if their rating was < 3 (i.e. the concreteness scale midpoint) and as concrete if their rating was ≥ 3. Sensorimotor distance was available for 993 of 999 Simlex concept pairs, which we then split as follows: 264 abstract–abstract pairs, 172 mixed pairs (i.e. one abstract, one concrete), and 557 concrete–concrete pairs.

### Analysis

We computed Bayesian correlations between sensorimotor distance and Simlex similarity judgements (JASP Team, 2020) with a stretched prior beta width = 1 (i.e. uniform prior where all correlations values are equally likely), and the alternative hypothesis that the variables would be correlated negatively (i.e. more similar = shorter distance). Correlations were computed separately for each category of concept pair.

### Results

Sensorimotor distance correlated with human similarity judgement comparably well for all categories of concept pair (see Fig. 8). The highest correlation was actually for mixed word pairs, but—importantly—the correlations for abstract–abstract pairs and concrete–concrete pairs were close in magnitude, and comparable given their 95% credible intervals. These results suggest that sensorimotor distance is a useful measure of semantic similarity for all concept pairs, abstract and concrete alike.

**Fig. 8:**
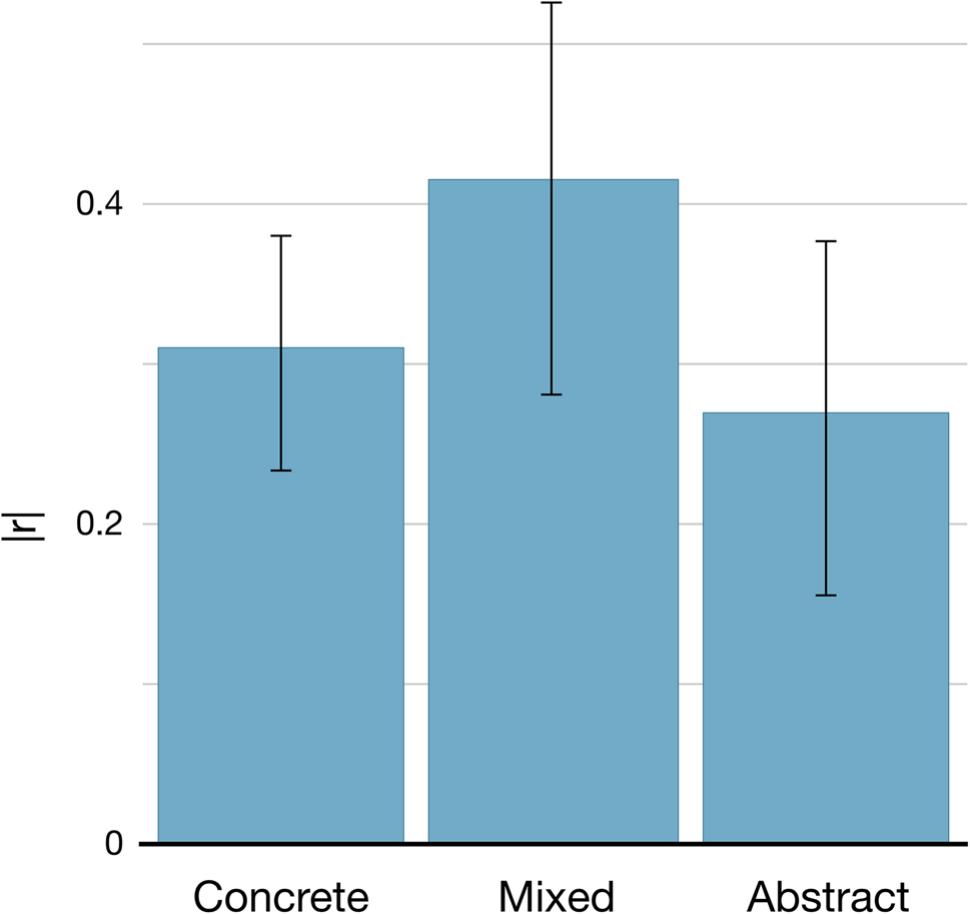
Absolute value of correlations between sensorimotor distance and human similarity judgement for the Simlex dataset, for word pairs where both are concrete, both are abstract, and mixed pairs. Error bars show the 95% credible intervals for the correlation value

## General discussion

We have presented sensorimotor distance, a novel grounded measure of semantic similarity for nearly 800 million concept pairs that is based on Lynott et al.'s (2020) 40,000-concept sensorimotor strength norms. Unlike existing measures of semantic similarity (e.g. CBOW, WordNet, feature overlap), sensorimotor distance directly operationalises sensorimotor experience in multiple perceptual modalities and action effectors, and is therefore grounded in how it constrains similarity. The semantic information represented by sensorimotor distance is transparent, relevant to all concepts/words regardless of their concreteness or grammatical class, and available at a scale that covers a full-sized adult conceptual system for a native speaker of English.

In validating sensorimotor distance, we demonstrated that it captures information about semantic similarity that is not captured by alternative measures, and that human judgements of similarity are best fit by combining multiple similarity measures in a single model. Indeed, the optimal combination of similarity measures varied markedly from one dataset to the next, which highlights the importance of validating semantic similarity measures against multiple human benchmarks, yet sensorimotor distance was the most consistent predictor across datasets. These findings support the idea that, when people judge if things are semantically similar, they employ multiple constraints on what similarity might mean. Multiple measures of similarity, each applying different constraints, are therefore required to fully capture the similarity between two given concepts (see Goodman, 1972).

Like many semantic predictors used in cognitive psychology (including some other predictors used in this study: feature overlap and taxonomic distance), sensorimotor distance is ultimately derived from participant responses in a task which involves access to words’ semantic representations. Therefore, insofar as such a predictor is used to model cognitive processes or representations which themselves involve accessing word semantics—as is common in the cognitive sciences—it cannot account for the dereferencing of mental concepts from their labels per se (Westbury, 2016; Wittgenstein, 1953). In theory, one might hope to derive the multidimensional vector from direct recordings of activation in participants’ sensorimotor cortices (e.g. Hauk et al., 2004) while they experience (and recall, name, etc.) various concepts across various contexts, and to use these recordings to quantify the degree to which different perceptual modalities and action effectors were involved in direct experience of each particular concept. Such measurements—and any resulting distance calculations between concepts—would qualify as an out-of-domain explanation of (part of) word semantics that would satisfy Westbury’s (2016) concerns about dormitivity. Of course, in reality, it would be completely impractical to conduct this hypothetical norming study at the scale of tens of thousands of concepts that comprise the human conceptual system (e.g. Hauk et al. required high-resolution functional and structural magnetic resonance imaging (MRI) scans to localise 14 participants’ responses to 150 test words). Instead, the measures that underlie sensorimotor distance (i.e. the Lancaster Sensorimotor Norms), as explained by Lynott et al. (2020), aim to approximate it via introspective judgements of sensorimotor experience. We believe that by restricting the domain of judgement so tightly, the Lancaster Sensorimotor Norms provide a reasonable proxy for direct sensorimotor experience (see also Reilly et al., 2020) in a tractable way, as well as allowing the pool of items to easily extend to traditionally abstract and/or physically diffuse concepts (e.g. *democracy*, which is perhaps easier to characterise through introspection than to experience in a lab setting) that nonetheless appear to have a robust, situated, sensorimotor grounding. Sensorimotor distance, based on this reasonable proxy of sensorimotor experience, therefore provides a tractable operationalisation of how the distributed representations of two concepts across perception and action systems differ from one another.

Of course, the particular 11 dimensions that we use here to calculate sensorimotor distance are not the only possible way to specify dimensions of perception and action experience. Although each dimension is well motivated (see Lynott et al., 2020, for details), they exhibit a complex intercorrelational structure that corresponds to how the human body’s senses and effectors interact with the external world. This structure reflects, for example, that things which can be touched can usually be seen, or that things which can be tasted can usually also be smelled but are not usually subject to action with the foot/leg. As a result, one might be concerned that some dimensions are redundant, and that cosine distance therefore produces a skewed picture of what sensorimotor distance should reflect. However, cosine distance (which is sensitive to this correlated structure) overall *outperforms* Mahalonobis distance (which removes this correlated structure: Mahalanobis, 1936; see supplementary materials for full results), which suggests that the present 11-dimensional space is a reasonably accurate reflection of how sensorimotor information informs human judgements of semantic similarity. Nonetheless, Mahalonobis distance is available in the web tool for researchers who explicitly wish to use it^8^. Alternatively, one may wonder if more fine-grained distinctions of sensorimotor experience would be useful, so long as they still meet the same criteria as the original dimensions (i.e. perception or action experience that is processed in a distinct cortical region). For example, visual perception could be subdivided into colour versus visuospatial movement, haptic perception could be subdivided into sensation on the hand versus elsewhere on the body, hand/arm action could be subdivided into action of the hand versus the arm/shoulder area, and so on. Whether such fine-grained distinctions would help or hinder the accuracy of sensorimotor distance in predicting semantic similarity remains an open question for future research.

### Conclusion

We hope that sensorimotor distance, available in an online application at https://lancaster.ac.uk/psychology/smdistance (see Appendix 1), will provide a useful tool for researchers in cognitive psychology, psycholinguistics, cognitive neuroscience, or any field relevant to semantic similarity and the grounded nature of concepts in semantic memory.

## Figures and Tables

**Fig. 9:**
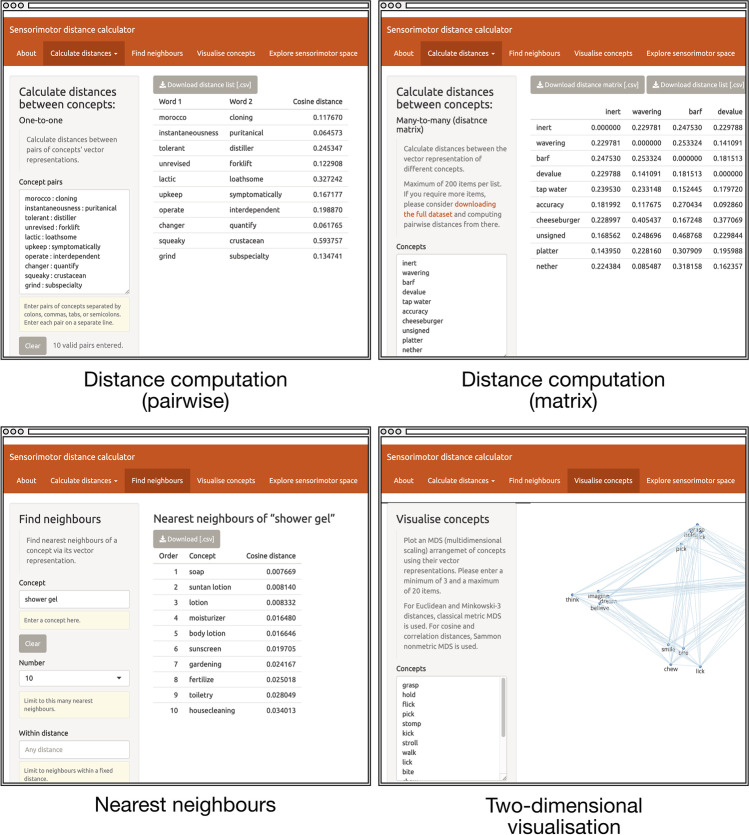
Four sample modes of the sensorimotor distance online interface, showing (clockwise from top left): computing distance between pairs of words; computing distance matrices between lists of words; visualizing sensorimotor distance between concepts in two dimensions; finding nearest neighbours

**Fig. 10:**
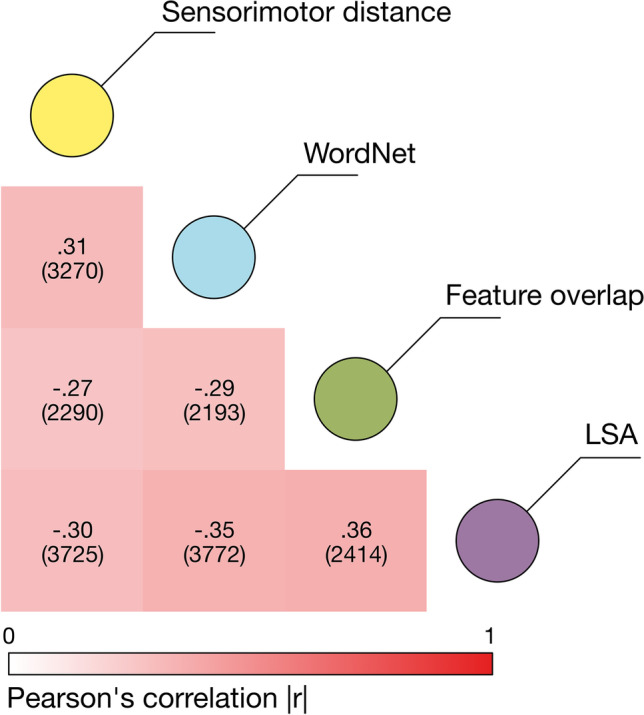
Correlations between sensorimotor distance and three other measures of semantic similarity. The colour scale indicates the absolute value of the correlation (i.e. stronger colour = stronger relationship) while the correlation sign varies according to whether the variable is a measure of distance or similarity/overlap. The number of pairs per comparison is in parentheses (2081 pairs were common to all measures). (See Fig. 4 for comparison with CBOW.)

**Fig. 11:**
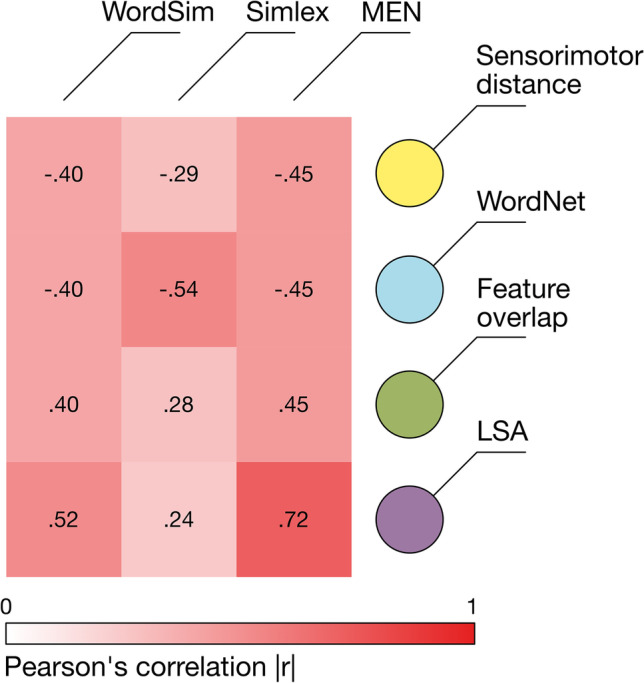
Zero-order correlations between human similarity judgements and each measure of semantic similarity, calculated separately per dataset. The colour scale indicates the absolute value of the correlation (i.e. stronger colour = stronger relationship) while the correlation sign varies according to whether the variable is a measure of distance or similarity/overlap. (See Fig. 5 for a comparison with CBOW.)

**Fig. 12:**
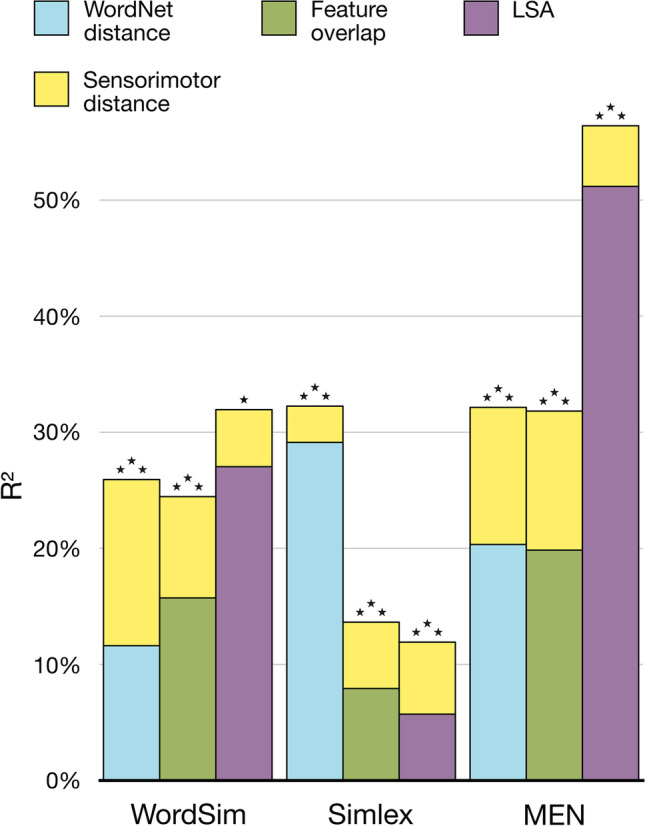
Unique effects of sensorimotor distance (top of stacked bar; yellow) in explaining variance in human similarity judgements when added to a regression model already containing an alternative measure of semantic similarity (bottom of stacked bar; colour varies). Regressions were performed separately for each dataset (WordSim, Simlex, MEN) and alternative measure of semantic similarity (WordNet distance, feature overlap, LSA). Asterisks indicate evidence for including sensorimotor distance at Step 2 compared to alternative predictor at Step 1 (*: log BF_10_> log 10, **: log BF_10_> log 100, ***: log BF_10_ > log 1000). (See Fig. 6 for a comparison with CBOW.)

**Fig. 13:**
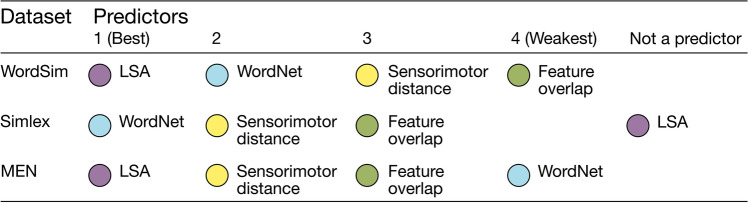
Rank order from best to worst of each semantic similarity measure in predicting human similarity judgements across three datasets, based on inclusion Bayes factors in best-model regressions. (See Fig. 7 for a comparison with CBOW.)

## Data Availability

All images, code, and data used in this article are available at https://osf.io/d42q6/, licensed under a Creative Commons Attribution 4.0 International License (CC-BY), which permits use, sharing, adaptation, distribution, and reproduction in any medium or format, so long as the user gives appropriate credit to the original authors and source, provides a link to the Creative Commons license, and indicates whether changes were made. Sensorimotor distance measures are available via a web tool at https://www.lancaster.ac.uk/psychology/smdistance/ under the same licence terms. To view a copy of the license, visit http://creativecommons.org/licenses/by/4.0/

## Appendix 1: Web tool for sensorimotor distance calculation

For convenience, sensorimotor distances and related operations can be computed using a web-based tool developed by the authors and available at https://www.lancaster.ac.uk/psychology/smdistance/. The available functions include calculating sensorimotor distance between concepts (pairwise, one-to-many, or many-to-many matrix), producing a list of nearest neighbours in sensorimotor space for a given concept, and visualizing two-dimensional representations of sensorimotor distance between concepts. Appendix Fig. 9 illustrates some examples of the interface.

## Appendix 2: A comparison with latent semantic analysis

Latent semantic analysis (LSA: Landauer & Dumais, 1997) continues to have extremely widespread use in the cognitive sciences as a measure of semantic similarity (e.g. Dautriche et al., 2017; Gagné et al., 2020; Ren & Coutanche, 2021). We examined LSA as an alternative linguistic distributional model to CBOW. Overall, LSA was a somewhat worse predictor of human similarity judgements than CBOW, but it exhibited the same qualitative patterns. Critically, it exhibited a very similar pattern relative to the other predictors, and in particular relative to sensorimotor distance. We include the relevant figures of results below (Appendix Figs. 10, 11, 12 and 13); full details are available in the supplemental materials^9^ (https://osf.io/d42q6/).

## Footnotes

1 e.g. *alligator → crocodilian reptile → diapsid reptile → reptile → vertebrate → chordate → animal → organism → living thing → whole → physical object → physical entity → entity → abstract entity → psychological feature → cognition → ability → creativity → imagination → imaginary being → monster*.

2 Some recent distributional models incorporate co-occurrence of either visual or auditory information as well as linguistic (e.g. Bruni et al., 2014; Günther et al., 2020; Lazaridou et al., 2015; Lopopolo & van Miltenburg, 2015), but these models fall outside the linguistic domain of the distributional hypothesis, and they are not currently widely used in cognitive psychology to approximate semantic similarity.

3 We note that sensorimotor grounding of word meaning can occur not only via direct, first-hand experience, but also indirectly via vicarious experience or inference from linguistic associations (Barsalou, 1999; Connell & Lynott, 2014; Harnad, 1990; Louwerse, 2011). Because each concept’s vector operationalises grounding in sensorimotor systems, regardless of how this grounding is acquired, it means that sensorimotor distance between concepts is also fully grounded in this way. Nonetheless, even though we use vector comparison as the basis for sensorimotor distance, we do not suggest that the mental processes underlying semantic similarity judgements actually resemble vector operations (Jones et al., 2015).

4 We opted to use cosine distance rather than cosine similarity for consistency with other distance measures in the web tool and to avoid terminological confusion with semantic similarity.

5 While Lynott et al. (2020) discussed some variables with similar names in the original Lancaster Sensorimotor Norms paper, they all compressed the multidimensional sensorimotor strength of *a single concept* by calculating the distance from a concept vector to the origin. By contrast, the measures we present here compare *two separate concepts* by calculating the distance between their individual vectors.

6 Buchanan et al. (2019) provide two measures of feature overlap using cosine distance: one using the original features provided by their participants ("raw" overlap), and another where features were combined using a root lemmatiser ("root" overlap). In what follows we use the "root" overlap measure.

7 There were 264 abstract–abstract pairs for which sensorimotor information was available (26% of dataset) in Simlex-999, but only 41 in WordSim-353 (11% of dataset) and 17 in MEN (<1% of dataset).

8 We thank Fritz Günther for this suggestion
